# Comparing functionality, anxiety and depression during pregnancy and postpartum in women with hypertensive disorders: Findings from the WOICE assessment

**DOI:** 10.1002/ijgo.71211

**Published:** 2026-07-05

**Authors:** Stephanie Pabón Lozano, José Paulo Siqueira Guida, Charles Mpoca Charles, Helen Johana Ortiz Rojas, Carolina Gómez Muñoz, Jose Guilherme Cecatti, Maria Laura Costa

**Affiliations:** ^1^ Department of Gynecology and Obstetrics University of Campinas Campinas Brazil; ^2^ Fundación Valle del Lili Healthcare Knowledge Management Cali Colombia; ^3^ Fundación Valle del Lili Clinical Research Center Cali Colombia

**Keywords:** anxiety, depression, functioning, postpartum care, prenatal care

## Abstract

**Objective:**

To describe the use of the WOICE tool for assessing non‐severe maternal morbidities among pregnant and postpartum women with hypertensive disorders.

**Methods:**

Secondary analysis of a previously published cross‐sectional study performed in Brazil, with the inclusion of women with hypertensive disorders (pre‐eclampsia, chronic hypertension, and gestational hypertension), comparing them in the antenatal and postpartum periods. The WOICE tool was applied during 28 weeks of gestational age and in the 40th day after delivery. The study was conducted at a referral maternity hospital between November 2017 and December 2018. Descriptive and comparative analyses were performed using appropriate statistical tests. Prevalence ratio (PR) identified factors associated with functional impairment were assessed through multivariable analysis.

**Results:**

Of 1046 women, 238 had hypertensive disorders and were included (99 pregnant, 139 postpartum). The mean age was 30 years old in both groups. Most participants had completed secondary education and were married. Among women in the postpartum period, there was a significant decrease in the frequency of employed women (*P* = 0.009). Functional impairment was reported in 40.4% versus 12.2% (*P* < 0.001); anxiety affected 31.3% versus 14.4% (*P* < 0.001); depressive symptoms were present in 13.1% versus 4.3% (*P* < 0.001), respectively, ante‐ and postpartum. Substance use was similar (13.1% vs. 14.4%). Risk of violence was reported by 7.1% of pregnant and 5.0% of postpartum women (*P* = 0.581). Having pre‐existing conditions (PR 2.40, confidence interval [CI]: 1.28–4.51, *P* = 0.006) and anxiety (PR 2.75, CI: 1.31–5.77, *P* = 0.008) were associated with impaired functioning in prenatal care. Breastfeeding protected women from impaired functioning in the postpartum period (PR 0.12, CI: 0.02–0.69, *P* = 0.017), while having pre‐existing conditions (PR 4.40, CI: 2.29–8.74, *P* < 0.001) increased the risk during the postpartum period.

**Conclusion:**

Anxiety, depression, and functional impairment were frequent among women with hypertensive disorders during pregnancy, occurring during antenatal and postpartum care. These findings highlight the need of routine maternal mental health screening and multidisciplinary care to identify and properly treat women affected by those conditions.

## 1 INTRODUCTION

Hypertensive disorders are among the leading causes of maternal morbidity and mortality, with 16% of all maternal deaths worldwide attributed to these conditions, while in Latin America and the Caribbean, this is estimated at 22%.^1^ These disorders represent a diverse group that includes chronic hypertension, gestational hypertension, and pre‐eclampsia, and these can complicate up to 10% of all pregnancies.^2^, ^3^ Antenatal care of women with hypertensive disorders demands close maternal and perinatal surveillance, because of the risks of adverse outcomes for both mother and fetus.^4^, ^5^

For more than a decade, severe maternal morbidity and maternal deaths have been extensively studied based on clinical definitions and specific criteria for case identification. This approach encompasses a range of conditions, from potentially life‐threatening conditions (PLTC) to maternal near miss (MNM), as established by the WHO.^6^

For every maternal death, 20–30 women survive pregnancy‐related morbidities, with possible repercussions on their abilities to perform daily activities, care for themselves, and/or interact independently with society. However, estimates are based on different methodologies, which are not always standardized.^7^

Regarding non‐severe maternal morbidity, WHO defined maternal morbidity as “any health condition attributed to and/or aggravated by pregnancy and childbirth that negatively impacts a woman's well‐being.”^8^ Based on this definition, WHO developed a tool to understand the impact of non‐severe morbidity in the obstetric population. This tool integrates various validated instruments to assess conditions such as depression, anxiety, functionality, drug use, exposure to violence and satisfaction with sexual life, aiming to provide a comprehensive understanding of women's health beyond severe complications. The tool was named WOICE: WHO Maternal Morbidity Tool.^9^

The WOICE tool was initially pilot‐tested in three different countries (Jamaica, Kenya, and Malawi)^9^ and then applied in Brazil.^10^, ^11^ However, it has not been evaluated in women with comorbidities, such as hypertension, to assess how these influence women during pregnancy and the postpartum period.

Although the clinical consequences of hypertensive disorders have been extensively investigated, less attention has been given to their broader impact on women's functioning, mental health, and emotional well‐being. The lack of systematized mental health screening during pregnancy and postpartum may underestimate the magnitude of the problem screened by the WOICE tool.^12^ Women with hypertensive disorders face a more traumatic experience during pregnancy and postpartum,^13^ with a higher prevalence of depression, with an important influence of personal history of mental illness.^14^ Pre‐eclampsia was associated with postpartum depression and postpartum psychosis.^15^

Several biological and psychosocial pathways may explain the impact of hypertensive disorders on women's functioning and mental health. Hypertensive disorders complications, especially pre‐eclampsia, are driven by with systemic inflammation and endothelial dysfunction, determining physical symptoms, frequent medical interventions, and the risk of incremented maternal and fetal complications.^16^, ^17^ All these factors may contribute to worsening women's experience during pregnancy, causing fatigue, physical limitations, and psychological distress.

The fear of disease progression, with the risk of preterm birth, the concern about fetal distress, and the complications to own health, may generate substantial emotional burden during pregnancy and the postpartum period. All these interlinked biological and psychosocial factors may negatively influence women's experience during pregnancy and postpartum.^18^

These interrelated pathways are consistent with the biopsychosocial framework underpinning the WHO's WOICE holistic approach. The use of the WOICE comprises multidimensional assessment, evaluating health domains that may not be identified through conventional obstetric indicators alone. Most importantly, we proposed in this secondary analysis the comparison of women with hypertensive disorders during pregnancy and after delivery, aiming to evaluate overall health in both periods and identify factors associated with impaired functioning.

## 2 MATERIALS AND METHODS

### 2.1 Study design

This was a secondary analysis of a cross‐sectional study conducted from November 2017 to December 2018 among women during antenatal (*N* = 531) and postpartum (*N* = 515) care at the maternity hospital of the University of Campinas, in southeast Brazil. The overall results from each arm of this study have already been published separately elsewhere.^10^, ^11^, ^19^ For this analysis, we included only women who had hypertensive disorders.

The WOICE tool includes different instruments that assess non‐severe morbidities among pregnant and postpartum women. The tool consists of several previously validated scales. To assess functionality and ability to perform daily tasks, the WHO Disability Assessment Schedule 2.0–12 item (WHODAS 2.0 12‐item version) was used. The WHODAS 2.0 12‐item version can assess six domains of life: cognition, mobility, self‐care, life activities, participation, and relationship with people.

Anxiety and depression were assessed using the Generalized Anxiety Disorder 7‐item (GAD‐7) and Patient Health Questionnaire 9‐item (PHQ‐9). To measure substance use, sexual satisfaction and exposure to violence, parts of some already validated scales were used, such as the Alcohol, Smoking and Substance Use Screening Test (ASSIST) and the Brief Sexual Symptom Checklist for Women (BSSC‐W), including some questions from a questionnaire used in the WHO multicenter study on women's health and domestic violence against women.

### 2.2 Setting

The facility where the study was implemented is a tertiary maternity hospital covering a metropolitan area of approximately 3 million inhabitants in the Brazilian southeast region. It provides high‐risk obstetric care. Weekly, the antenatal care outpatient clinic sees around 150 women, while 30 women attend the postpartum consultation. The multidisciplinary team includes obstetricians, nurses, physiotherapists, psychologists, nutritionists, social workers, and other health professionals.

The maternity hospital provides delivery care to low‐ and high‐risk pregnancies, attending around 200 childbirths monthly. It has a 40‐bed obstetric unit admitting pregnant and postpartum women, and a neonatal intensive care unit with 15 beds, and a neonatal semi‐intensive unit with 15 beds.

### 2.3 Study population

Women who received routine care at the institution and met the inclusion criteria: pregnant women from 28 weeks' gestation and postpartum women from 6 to 12 weeks postpartum. To identify women included in this analysis, we selected those with a confirmed diagnosis of chronic hypertension, gestational hypertension, or preeclampsia under the pre‐existing conditions assessment. The assessment of the conditions considered was conducted during data collection for the study, and performed to all women included in the study. No missing data was observed in the database of the study.

Hypertension during pregnancy was defined as systolic blood pressure (SBP) ≥140 mmHg and/or a diastolic blood pressure (DBP) ≥90 mmHg, on two occasions at least 4 h apart, or an isolated measurement of SBP ≥160 mmHg and/or DBP ≥110 mmHg. For pre‐eclampsia, the definition considered hypertension and organ dysfunction, including proteinuria or another target‐organ lesion. Proteinuria was defined by a 24‐h urine collection higher than 300 mg, or a protein/creatinine ratio higher than or equal to 0.3 in a urine spot. Target‐organ lesions included occurrence of hemolysis, liver dysfunction, kidney dysfunction, or symptoms such as headache, abdominal pain, or visual symptoms.

Hypertension diagnosed before 20 weeks of pregnancy was defined as chronic hypertension, while hypertension diagnosed after 20 weeks of pregnancy with no sign of organ dysfunction was defined as gestational hypertension.

The identification of hypertension and the classification of hypertensive disorders were performed by trained researchers (two nurses and one physician) who were specifically trained prior to data collection. The classification was included in the data collection sheets; dubious cases were prospectively discussed with the research supervisors to clarify them.

### 2.4 Data collection

A trained researcher administered the WOICE questionnaire to each participant after informed consent was signed. The data was entered into an online database using the REDCap software via tablets, with a server at the University of Campinas, where data protection and storage is ensured.

The data collection period was from November 2017 to December 2018. After the data collection was completed, the information was downloaded in a format compatible with the statistical software used. All data were reviewed to assess consistency. Checking and corrections were performed whenever needed.

### 2.5 Data analysis

To carry out this analysis, we identified and selected participants with hypertensive disorders from the original cohort of 1046 women. A total of 238 women met the inclusion criteria: 99 pregnant women (≥28 weeks' gestation) and 139 postpartum women (6–12 weeks' postpartum). These two groups were defined for comparative analysis.

A descriptive analysis of sociodemographic and clinical characteristics of the study population was performed, as well as the prevalence of non‐severe maternal morbidity conditions assessed by the WOICE instrument.

Functionality or abilities to perform activities of daily living were assessed with the WHODAS‐12, for which the 95th percentile was considered as the cutoff point to identify functionality impairment, according to a previous study that evaluated a similar population during the postpartum period, with a score ≥37.4.^20^ For anxiety and depression conditions, the GAD‐7 and PHQ‐9 scores were considered, where a score ≥10 indicated anxiety and/or depression, respectively.^17^, ^18^

The general sociodemographic characteristics and the scores for each condition assessed were compared between both groups using Student's *t*‐test or Chi‐square tests as appropriate.

A multiple analysis was conducted to evaluate factors associated with functional impairment generating adjusted prevalence ratios (PR) with 95% confidence interval (CI) for each group. Associations between independent variables and the outcomes were evaluated using Poisson regression models with robust variance. This approach was selected because the study had a cross‐sectional design and aimed to estimate PR, which are more interpretable than odds ratios when outcomes are frequent.

This analysis was carried out using two separate models, one for the prenatal group and another for the postpartum group. The outcome in both models was a WHODAS score ≥37.4 and the predictors evaluated were the variables age, educational level, marital status, occupation, time of transfer to the health institution, parity, rating of health status, pre‐existing conditions, referring any clinical condition, requiring referral to a service, anxiety (GAD‐7 ≥10), depression (PHQ‐9 ≥10), body mass index (BMI, calculated as weight in kilograms divided by the square of height in meters) (≥30). For the postpartum group, we also included breastfeeding as a variable in the model. The variables included in the multiple analysis were chosen by the researchers based on their clinical significance.

For statistical analysis, we adopted the software RStudio and R4.4.1.

### 2.6 Ethical considerations

The study was approved by the local Institutional Review Board of the University of Campinas (UNICAMP) (no.: 78497817.0.0000.5404). All participants signed an informed consent before the interview.

## 3 RESULTS

In the original study, 1046 women were included. In this analysis, only women with hypertensive disorders were selected, comprising a total of 238 women. Of them, 139 were in the postpartum group and 99 were in the prenatal care group. Figure 1 presents the flow chart of the included women.

**FIGURE 1:**
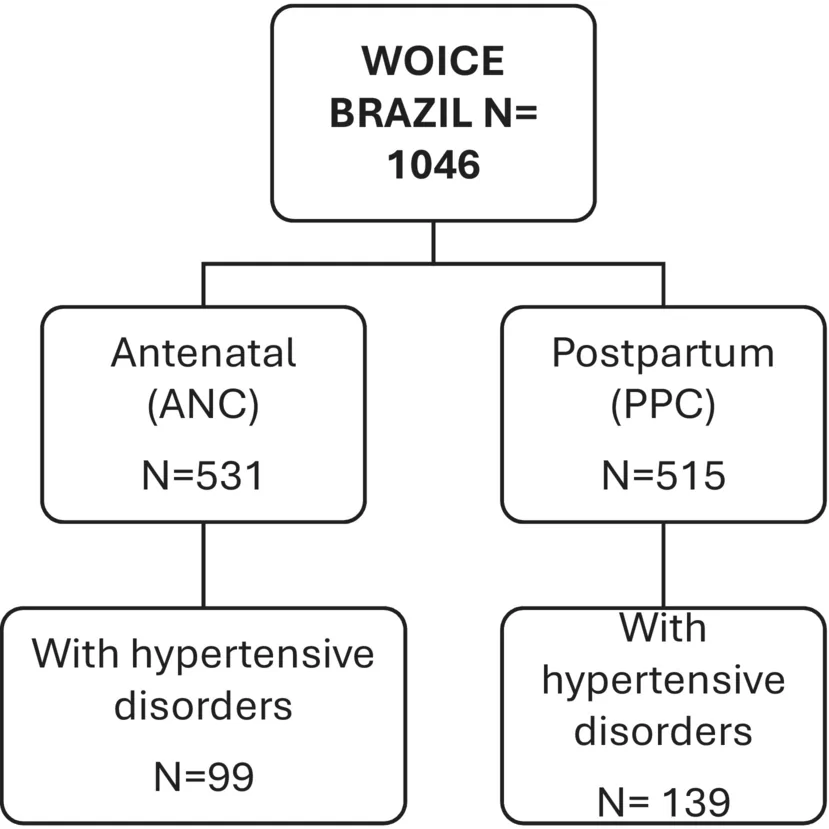
Flow chart of inclusion in this study.

Of the 99 women included in antenatal care, 39 (39.4%) had gestational hypertension, 16 (16.2%) had pre‐eclampsia, and 44 (44.4%) had chronic hypertension. Of the 139 postpartum women included, 24 (17.3%) had gestational hypertension, 35 (25.2%) had pre‐eclampsia, and 80 (57.5%) had chronic hypertension. The distribution of hypertensive disorders differed significantly between groups, with a higher proportion of gestational hypertension in antenatal care (*P* < 0.001).

The mean age of pregnant women was 31.1 (±6.2) years, and that of postpartum women was 30.3 (±7.0) years (*P* = 0.363). In both groups, most women had secondary education (*P* = 0.574) and were with a partner (*P* = 0.289). Among the pregnant women included, 20 (20.2%) were in their first pregnancy, while 28 (28.2%) were in their third or subsequent pregnancy (*P* = 0.041). In the postpartum group, nearly one‐third had their first delivery, while 31 (22.3%) reported having had three or more deliveries (*P* = 0.041). More than half of postpartum women had a cesarean delivery (77.0%), and 39.6% experienced preterm births, with a high rate of breastfeeding among them (91.2%). Additionally, most of the women reported having an occupation (73.7%) in the prenatal group, and 56.8% in the postpartum group. Table 1 presents the sociodemographic characteristics of the included women.

**TABLE 1:** Demographic characteristics of the women with hypertensive disorders included in the analysis.

Variable	Prenatal care	Postpartum care	*P* value
*N* = 238	99	139	
Age (mean, SD)	31.1 (±6.2)	30.3 (±7.0)	0.363
Education level			0.574
None	1 (1.0%)	3 (2.2%)	
Primary	23 (23.2%)	24 (17.3%)	
Secondary	64 (64.6%)	92 (66.2%)	
University	11 (11.1%)	20 (14.4%)	
Marital status			0.289
With partner	78 (78.8%)	100 (71.9%)	
Without partner	21 (21.2%)	39 (28.1%)	
Parity			**0.041**
0	20 (20.2%)	49 (35.3%)	
1–2	51 (51.6%)	59 (42.4%)	
≥3	28 (28.2%)	31 (22.3%)	
Cesarean	‐	107 (76.9%)	
Preterm birth	‐	55 (39.6%)	
Breastfeeding	‐	124 (91.2%)	
Occupation			**0.009**
Yes	73 (73.7%)	79 (56.8%)	
Not	26 (26.3%)	60 (43.2%)	

*Note*: *P*‐values lower than 0.05 were considered statistically significant, and were highlighted in bold.

Abbreviation: SD, standard deviation.

Among the included pregnant women, 47.5% of them rated their health as good, 31.3% rated it as neither good nor bad, and 12.1% rated their health as “bad” or “very bad.” Regarding those in the postpartum period, more than half of the women with hypertensive disorders (57.6%) rated their health as good, and only 7.2% considered their health to be “bad” or “very bad.” These results are presented in Table 2.

**TABLE 2:** Self‐perception of health, functionality and mental health among pregnant Brazilian women with hypertensive disorders according to the WOICE tool.

Variable	Prenatal care (*n* = 99)	Postpartum care (*n* = 139)	*P* value
Overall self‐perception of health			**<0.001**
Very good	9 (9.1%)	32 (23.0%)	
Good	47 (47.5%)	80 (57.6%)	
Neither good nor bad	31 (31.3%)	17 (12.2%)	
Bad	10 (10.1%)	9 (6.5%)	
Very bad	2 (2.0%)	1 (0.7%)	
WHODAS			
Mean score	37.1 (±13.5)	26.7 (±8.3)	**<0.001**
WHODAS ≥37.4	40 (40.4%)	17 (12.2%)	**<0.001**
Domains			
D1 (cognition)	2.9 (±1.4)	2.4 (±1.0)	**0.001**
D2 (mobility)	5.1 (±2.2)	3.1 (±1.6)	**<0.001**
D3 (self care)	3.9 (±1.9)	2.3 (±1.1)	**<0.001**
D4 (relationship with people)	2.5 (±1.4)	2.3 (±1.0)	0.200
D5 (life activities)	3.9 (±2.0)	2.9 (±1.5)	**<0.001**
D6 (participation)	3.8 (±2.1)	3.1 (±1.4)	**0.002**
Number of days in the last month that daily life activities were affected	20.5 (±11.0)	13.7 (±12.3)	**<0.001**
Number of days in the last month completely unable to perform daily tasks	1.8 (±4.4)	1.1 (±3.4)	0.167
Number of days in the past month that daily living tasks were reduced due to health problems	10.7 (±12.3)	3.9 (±8.7)	**<0.001**
Anxiety			
Mean score	7.1 (±6.5)	4.4 (±5.1)	**<0.001**
Levels of anxiety			0.003
No anxiety (0–4)	46 (46.5%)	91 (65.5%)	
Mild anxiety (5–9)	22 (22.2%)	28 (20.1%)	
Severe anxiety (≥10)	31 (31.3%)	20 (14.4%)	
Depression			
Mean score	5.1 (±6.7)	6.1 (±3.2)	0.126
Levels of depression			**<0.001**
No depression (0–4)	58 (58.6%)	13 (9.4%)	
Mild depression (5–9)	28 (28.2%)	120 (86.3%)	
Severe depression (≥10)	13 (13.1%)	6 (4.3%)	

*Note*: *P*‐values lower than 0.05 were considered statistically significant, and were highlighted in bold.

Abbreviation: WHODAS, WHO Disability Assessment Schedule.

Table 2 presents the results regarding functional impairment and mental health issues of the included women. During pregnancy, the mean WHODAS score was 37.1 ± 13.5, while the postpartum group had a lower score (26.7 ± 8.3), with a *P* < 0.001. It was identified that 40 pregnant women (40.4%) and 17 postpartum women (12.2%) had a functional impairment or their ability to perform activities of daily living affected (*P* < 0.001).

Table 2 also presents the WHODAS evaluation by domain. We observed that there was a decrease in mean score in five of the six domains considered (cognition, mobility, self care, life activities, and participation). The mean score remained stable only for relationship with people.

The women's perception of the number of days in the last month when functionality was impaired was 20.5 ± 11.0 for the antenatal group and 13.7 ± 12.3 for the postpartum group (*P* < 0.001), while the number of days in the last month when they were totally unable to perform their daily activities was 1.8 ± 4.4 and 1.1 ± 3.4, respectively (*P* = 0.167).

The mean GAD‐7 score was 7.1 ± 6.5 in the pregnant group and 4.4 ± 5.1 in the postpartum group (*P* = <0.001). During pregnancy, 31 (31.3%) women had a score greater than 10; in the postpartum population, this percentage decreased to 14.4% (*P* = 0.003).

The average PHQ‐9 scores for depression remained relatively stable during pregnancy (5.1 ± 6.7) and postpartum (6.1 ± 3.2); *P* = 0.126. Among the pregnant women, 28.2% had mild depression (score 5–9), compared to 86.3% of postpartum women (*P* < 0.001), while 13.1% of pregnant women and 4.3% of postpartum women had scores ≥10 for depression (*P* < 0.001).

The use of substances was assessed by asking participants if they used cigarettes, alcoholic beverages, marijuana, inhalants, sedatives or sleeping pills, hallucinogens, opioids, and/or injectable drugs for non‐medical purposes (Table 3). It was found that the use of these substances was similar in both groups (13.1 and 14.4%, respectively, *P* = 0.850). Additionally, women were asked whether, during pregnancy or postpartum (depending on the group), any friend, family member, or other person expressed concern about their substance use. A frequency of 10.1% and 12.2% were identified, respectively (*P* = 0.682). Regarding the question about whether they had tried to reduce or stop using any substance, only 8.0% of the women in antenatal care and 10.0% of the women in the postpartum period attempted to decrease their consumption (*P* = 0.656).

**TABLE 3:** Risk of violence and drug use during prenatal and postpartum care among pregnant Brazilian women with hypertensive disorders according to the WOICE tool.

Variable	Prenatal care (*n* = 99)	Postpartum care (*n* = 139)	*P* value
Active sexual life	82 (82.8%)	43 (30.9%)	**<0.001**
Satistfaction with sexual life	76 (92.7%)	37 (86.0%)	0.360
Drugs use	13 (13.1%)	20 (14.4%)	0.850
Preoccupation of others with the use of drugs	10 (10.1%)	17 (12.2%)	0.682
Attempted to stop or reduce use of drugs	8 (8.0%)	14 (10.0)	0.656
Risk of intimate partner violence	7 (7.1%)	7 (5.0%)	0.581
Suffered physical violence	3 (3.0%)	2 (1.4%)	0.651

*Note*: *P*‐values lower than 0.05 were considered statistically significant, and were highlighted in bold.

The frequency of women with hypertensive disorders who were at risk of intimate partner violence during both periods was 7.1% in the prenatal period and 5.0% in the postpartum period (*P* = 0.581). This was identified through positive responses to the question of “whether they were afraid of their current partner/most recent spouse or anyone else.” Overall, 4.4% of women with hypertensive disorders reported experiencing physical violence; three women (3.0%) in the prenatal group and two women (1.4%) in the postpartum group. This was assessed through the question of whether they had ever been pushed, hit, or kicked by their spouse or anyone else. Women in the prenatal period did not report sexual violence, unlike the postpartum period, where one woman reported being forced by her partner to have sexual intercourse when she did not want to. These data are presented in Table 3.

Regarding sexual function, 82 (82.8%) women had sexual activity during pregnancy, and 76 (92.7%) were satisfied with their sexual life. In the postpartum group, 43 (30.9%) women had resumed sexual activities, and 37 (86.0%) were satisfied. Those results highlighted that the frequency of sexual activities was higher during prenatal care (*P* < 0.001), however, the satisfaction with the sexual life was similar in both groups (*P* = 0.360). These data are presented in Table 3.

After administering the survey, additional support was offered, and it was found that 38% of pregnant women and 20.1% of postpartum women requested follow‐up care. Among these, the frequency of women who sought referral for psychological care was 37% in the prenatal period and 20.1% in the postpartum period.

In the multivariate analysis, the presence of pre‐existing conditions and symptoms of anxiety (GAD‐7 ≥10) during pregnancy were factors independently associated with functional impairment (PR 2.40, 95% CI: 1.28–4.51, *P* = 0.006; PR 2.75, 95% CI: 1.31–5.77, *P* = 0.008, respectively). In the postpartum population, breastfeeding acted as a protective factor (PR 0.12, 95% CI: 0.02–0.69, *P* = 0.017), and having pre‐existing conditions, as in the prenatal population, was associated with functional impairment (PR 4.40, 95% CI: 2.21–8.74, *P* < 0.001) (Table 4).

**TABLE 4:** Factors associated with impaired functionality in a group of pregnant and postpartum women with hypertensive disorders–multivariate analysis.

Variable	PR	95% CI p/PR	*P* value
(a) Model 1: Functional impairment (WHODAS ≥37.4) (pregnant group)			
Having pre‐existing conditions	2.40	1.28–4.51	*P* = 0.006
Anxiety (GAD ≥10)	2.75	1.31–5.77	*P* = 0.008
(b) Model 2: Functional impairment (WHODAS ≥37.4) (postpartum group)			
Having pre‐existing conditions	4.40	2.21–8.74	*P* < 0.001
Breastfeeding	0.12	0.02–0.69	*P* = 0.017

*Note*: BMI, calculated as weight in kilograms divided by the square of height in meters. Multiple regression analysis with multivariate Poisson models (robust SE): For both models a WHODAS score ≥37.4 was taken into account as an outcome and the predictors evaluated were the variables age, educational level, marital status, occupation, travel time to the health institution, parity, health status rating, pre‐existing conditions, refer any clinical condition, require referral to any service, anxiety (GAD‐7 ≥10), depression (PHQ‐9 ≥10), BMI (≥30).

Abbreviations: BMI, body mass index; CI, confidence interval; GAD‐7, Generalized Anxiety Disorder 7‐item; PHQ‐9, Patient Health Questionnaire 9‐item; PR, prevalence ratio; WHODAS, WHO Disability Assessment Schedule.

## 4 DISCUSSION

This study presents the results of a secondary analysis of the WOICE instrument, applied during antenatal and postpartum care in a referral maternity hospital in Brazil, with a total sample of 1046 women, of which 238 women with hypertensive disorders were identified, 99 during antenatal care and 139 in the postpartum period. The main results show that when comparing both groups, postpartum women presented lower frequencies of anxiety symptoms and functional impairment, but a higher frequency of mild depressive symptoms when compared with pregnant women.

The WOICE tool had previously been used in a pilot study in Jamaica, Kenya, and Malawi. Regarding sociodemographic data, the selected population for this analysis was older and had more than one pregnancy. Additionally, compared to the aforementioned study, our population had the similarity that most women had a partner, and mostly with a high school education.^21^ However, notable differences were observed, particularly regarding the frequency of cesarean deliveries, our study population showed a considerably higher rate compared to the pilot study (77.0% vs. 22.0%), which may be attributed to the increased frequency of high‐risk patients, with underlying medical conditions, including hypertensive disorders, that are associated with increased rates of cesarean delivery and a higher risk of preterm birth.^22^ In fact, the frequency of preterm births in our population was 39.6%, much higher than the baseline numbers for the population (around 12%).^23^

The WOICE measures functionality through the short version of WHODAS 2.0. A previous study conducted in a similar population identified that the best cutoff point for defining functional impairment with this instrument is the 95th percentile (score above 37.4 points, out of 100).^24^ During pregnancy, the mean WHODAS score for our study was 37.1 ± 13.5, decreasing to 26.7 ± 8.3 in the postpartum group. This contrasts with other studies conducted in Ghana and Côte d'Ivoire, where a mean WHODAS score of 13.0 ± 6.66 was found in Ghana and 11.8 ± 7.32 in Côte d'Ivoire. In general, these had a population of women with uncomplicated pregnancies, showing lower levels of dysfunction.^25^ Similar results were demonstrated in Santa Cruz, Brazil, evaluating women classified as low obstetric risk in the second and third trimesters with a WHODAS score of 10.7.^26^

Women with hypertensive disorders may present both, acute complications (medically indicated preterm birth, placental abruption, pulmonary edema, seizures, stroke, renal or hepatic dysfunction, among others) and long‐term complications (increased risk of cardiovascular disease, renal failure, neurological sequelae), which could lead to a negative impact on functionality. In our multivariate analysis, the presence of pre‐existing conditions was associated with impaired functionality in both pregnant and postpartum women. However, we hypothesize that during the first few weeks postpartum, the women's functioning assessment is associated with neonatal outcomes and that a positive outcome allows for improved functioning. This might account for the difference in considering findings during pregnancy, where the increased maternal and fetal surveillance places an extra burden that could be reflected also in functioning performance.^11^, ^27^

Additionally, the end of pregnancy itself may also play an important role in decreasing anxiety and improving functioning. The end of the physiological demands and uncertainties associated with pregnancy may contribute to these changes. Although our study did not directly assess women's priorities, the breastfeeding acted as a protective factor for functioning in the postpartum group which suggests an important focus on infant care. This is relevant in cases of premature or complicated births which were present in nearly 40% of postpartum women and may impact mothers' perceptions of functioning, mental health, and overall well‐being.

In our sample, around one third of pregnant women presented a score ≥10 on the GAD‐7 questionnaire for anxiety, similar to the frequency of anxiety in the primary study (29.9%).^11^ In contrast, a study carried out in Morocco about half of its population reported this condition.^20^

Regarding depression, the frequency among pregnant women in our study (13.1% of severe depression) was lower than that observed in the Moroccan study,^20^ where the frequency of this condition was 31%. Comparing the exposure to violence, we observed that, among Moroccan women, it reached 15.4%, while in our population, it was 15.4%. Exposure to stressors during pregnancy, such as intimate partner violence, is associated with higher levels of depression and anxiety.^22^, ^28^

The lower prevalence of severe depression observed among postpartum women should be interpreted cautiously. The proportion of women reporting mild depressive symptoms was higher, while the proportion of those without depressive symptoms was lower. This pattern suggests a redistribution of symptom severity rather than a true reduction in depressive burden. This is possibly explained by the onset of new challenges after delivery, such as infant care, sleep deprivation, breastfeeding difficulties, and adaptation to maternal responsibilities. Consequently, the postpartum period is a period of vulnerability to mental health issues, even if severe depression is less common.^29^, ^30^

Studies indicate that prenatal anxiety has been associated with adverse perinatal outcomes.^31^ Many social and economic factors have been related to depression and anxiety during pregnancy and postpartum, including the experience of a first pregnancy, whether the pregnancy is unwanted, exposure to partner violence, and lack of support.^9^ It is essential to identify and treat these conditions to improve well‐being, mental health, reduce the risk of postpartum depression, and decrease mortality and its effects on the child.

Anxiety emerged as an independent predictor of impaired functionality among women during prenatal care, similarly to a finding observed in a Chinese study.^32^ Pregnancy is a moment of women's life marked by changes that are physical, psychological, and in the relationship with family and community. The velocity of these changes, and the velocity of their occurrence, may be a trigger for the association between anxiety and impaired functioning observed in the studies.

During the COVID‐19 pandemic, the prevalence of depression and anxiety symptoms increased among women during pregnancy and the postpartum period.^33^ However, in this study, despite having been carried out before the pandemic, the frequency of anxiety in pregnant women was similar to that reported during the pandemic period.

Mental health challenges in the perinatal period are frequently related to other vulnerabilities, including substance use. In our study, 13.1% of pregnant women and 14.4% of postpartum women reported substance use, which was higher than the 0.4% reported in the Moroccan study.^20^ Substance use and dependency are among the most serious problems worldwide, with a high burden of morbidity. During pregnancy, substance use has negative consequences for both the mother and the fetus; pregnant women who use substances frequently have a history of inadequate antenatal and postnatal care and tend to be in a disadvantaged social situation, which predisposes them to adverse obstetric and perinatal outcomes, along with an increase in child abuse or neglect. Although the WOICE tool does not specify the exact substances consumed by women, the results show higher consumption percentages than those reported in other studies, indicating that this issue needs to be better addressed.

These overlapping vulnerabilities (mental health and substance use issues) can be worsened by experiences of violence, particularly intimate partner violence, a critical concern during the perinatal period. Violence against women is a public health issue and a violation of human rights. Its magnitude is such that, in the fifth Sustainable Development Goal of the United Nations, the importance of achieving gender equality, empowering women, and eliminating all forms of violence is addressed. The WHO estimates indicate that around one in three women globally has experienced physical and/or sexual partner violence or sexual violence by others at some point in their lives. Domestic violence during pregnancy can have physical and psychological consequences. A pregnant woman assaulted by her partner is at greater risk of suffering from stress, depression, and substance addiction.^34^

Our secondary analysis showed that 7.1% of women with hypertensive disorders in the prenatal period and 5.0% in the postpartum period had been exposed to some form of violence, compared to the pilot study conducted in Kenya, Malawi, and Jamaica, which reported 12.8%^9^ and 13.2% among Moroccan women.^20^ Our study likely obtained underestimated data since some women are often afraid to report violence due to possible repercussions, do not consider themselves victims of violence, or some forms of violence are culturally regarded as usual. Violence is difficult to identify and address, and it is necessary to have proper surveillance and provide spaces for women to express it so that the extent of the problem can be understood, and national policies can be proposed.

In our study, the most frequent causes of sexual dissatisfaction were lack of interest in sex and pain during intercourse. Sexual activity during pregnancy can be affected due to a combination of physical, emotional, and cultural factors.^44^ Sexual dysfunction increases as pregnancy progresses, and some people may believe that sexual intercourse during pregnancy can lead to adverse obstetric outcomes, harm the baby, or be considered unattractive or taboo. In the postpartum period, factors associated with sexual dissatisfaction include fatigue, stress, lack of sleep, focus on newborn care, hormonal changes, fear of pain, and body image perceptions.^35^, ^36^

One aspect to highlight is the high percentage of women who breastfed their children (91.2%), considering that these women have a high morbidity burden due to hypertensive disorders, along with the frequency of prematurity associated with this condition. This could reflect the active efforts of the hospital to educate women on the benefits of breastfeeding. According to a 2017 study, Brazil is considered a successful country in implementing policies and programs to promote breastfeeding.^37^

The observed protective association between breastfeeding and impaired functionality should be interpreted with caution. Breastfeeding may act as a marker of overall maternal health, since women with fewer physical limitations, lower psychological distress, and greater social support may be more likely to successfully breastfeed. Because the present study was cross‐sectional, it is not possible to establish temporality or infer causality. Therefore, the association observed may reflect both the influence of maternal functionality on breastfeeding practices and the potential benefits of breastfeeding on maternal well‐being.^38^

The WOICE instrument was administered after a scheduled antenatal care visit, and some of the questions may have been difficult, potentially triggering painful memories and revealing exposure to violence and substance abuse. After including participants in the survey, we provided additional support, including psychological follow‐up for the women. This was a strength because they could be referred to psychological care if needed.

Among the limitations of our study is the sample size. Since it was a secondary analysis to evaluate the results of WOICE in the population with hypertensive disorders, a significant part of the population evaluated in the primary studies was excluded. Furthermore, the questionnaire included some open‐ended questions, which did not have standardized responses. Additionally, it is not a questionnaire to evaluate comorbidities in detail, so the responses were not directed towards this purpose but rather to give voice to women's perceptions. It was also not possible to specify the type or severity of the hypertensive disorder in each case which may have different clinical impacts on maternal outcomes and functionality. This limits the intention to assess associations according to the specific hypertensive condition.

## 5 CONCLUSION

Women with hypertensive disorders of pregnancy exhibited high frequencies of anxiety, depression, and functional impairment during both pregnancy and the postpartum period, highlighting the substantial burden of non‐clinical morbidity in this population. These findings reinforce that women affected by hypertensive disorders represent a vulnerable group whose healthcare needs extend beyond blood pressure control and obstetric surveillance alone. Comprehensive maternal assessment, including routine screening of mental health, functioning, and psychosocial well‐being, should be incorporated into antenatal and postpartum care to support the early identification and management of conditions that may otherwise remain unrecognized. Expanding the focus of maternal care beyond traditional clinical outcomes may contribute to a more holistic and patient‐centered approach for women with hypertensive disorders.

## AUTHOR CONTRIBUTIONS

SPL performed data collection, reviewed data consistency, and wrote the first version of the draft. JPSG performed statistical analysis, reviewed the results, and wrote the first version of the draft. CMP performed data collection, reviewed data consistency, and reviewed the manuscript. HJOR reviewed data consistency and performed data analysis. CGM reviewed data consistency and performed data analysis. JGC reviewed the final manuscript. MLC had the original idea and reviewed the final manuscript. All authors approved the final version of the article.

## FUNDING INFORMATION

The authors have nothing to report.

## CONFLICT OF INTEREST STATEMENT

The authors confirm there are no conflict of interest.

## Data Availability

Data available on request due to privacy/ethical restrictions.
